# P34HB electrospun fibres promote bone regeneration in vivo

**DOI:** 10.1111/cpr.12601

**Published:** 2019-03-21

**Authors:** Na Fu, Zhaosong Meng, Tiejun Jiao, Xiaoding Luo, Zisheng Tang, Bofeng Zhu, Lei Sui, Xiaoxiao Cai

**Affiliations:** ^1^ School of Stomatology, Hospital of Stomatology Tianjin Medical University Tianjin China; ^2^ Department of Endodontics Shanghai Ninth People’s Hospital, College of Stomatology, Shanghai Jiao Tong University School of Medicine Shanghai China; ^3^ Key Laboratory of Shaanxi Province for Craniofacial Precision Medicine Research, College of Stomatology Xi’an Jiaotong University Xi’an China; ^4^ Clinical Research Center of Shaanxi Province for Dental and Maxillofacial Diseases, College of Stomatology Xi’an Jiaotong University Xi’an China; ^5^ Department of Forensic Genetics, School of Forensic Medicine Southern Medical University Guangzhou China; ^6^ State Key Laboratory of Oral Diseases, National Clinical Research Center for Oral Diseases West China Hospital of Stomatology, Sichuan University Chengdu China

**Keywords:** bone marrow mesenchymal stem cells, bone tissue engineering, calvarial defects, electrospinning, P34HB

## Abstract

**Objective:**

Bone tissue engineering was introduced in 1995 and provides a new way to reconstruct bone and repair bone defects. However, the design and fabrication of suitable bionic bone scaffolds are still challenging, and the ideal scaffolds in bone tissue engineering should have a three‐dimensional porous network, good biocompatibility, excellent biodegradability and so on. The purpose of our research was to investigate whether a bioplasticpoly3‐hydroxybutyrate4‐hydroxybutyrate (P34HB) electrospun fibre scaffold is conducive to the repair of bone defects, and whether it is a potential scaffold for bone tissue engineering.

**Materials and methods:**

The P34HB electrospun fibre scaffolds were prepared by electrospinning technology, and the surface morphology, hydrophilicity, mechanical properties and cytological behaviour of the scaffolds were tested. Furthermore, a calvarial defect model was created in rats, and through layer‐by‐layer paper‐stacking technology, the P34HB electrospun fibre scaffolds were implanted into the calvarial defect area and their effect on bone repair was evaluated.

**Results:**

The results showed that the P34HB electrospun fibre scaffolds are interwoven with several fibres and have good porosity, physical properties and chemical properties and can promote cell adhesion and proliferation with no cytotoxicity in vitro. In addition, the P34HB electrospun fibre scaffolds can promote the repair of calvarial defects in vivo.

**Conclusions:**

These results demonstrated that the P34HB electrospun fibre scaffold has a three‐dimensional porous network with good biocompatibility, excellent biosafety and ability for bone regeneration and repair; thus, the P34HB electrospun fibre scaffold is a potential scaffold for bone tissue engineering.

## 1 INTRODUCTION

Bone defects are a common clinical; at the present, there are common bone defects caused by congenital dysplasia, trauma and infection, and compressive fractures caused by senile osteoporosis, resection of bone tumours and revision afterarthroplasty.^1^, ^2^ The treatment of bone defects^3^ has always been one of a clinical challenge. The emergence of bone tissue engineering^4^ provides a new idea for repairing bone defects and achieves good results. Bone tissue engineering is a technical method of transferring cultured osteoblasts to scaffold materials and culturing the osteoblasts to form mineralized bone in vitro. Its basic principle is to implant osteoblasts cultured in vitro as seed cells into three‐dimensional scaffolds that have good biocompatibility and biodegradability and then implant the cell/scaffold complex in vivo or continue to cultivate the complex in vitro. After proliferation, differentiation and secretion of extracellular matrix, the cells form mineralized bone tissue. At the same time, the structure of the scaffolds is gradually degraded and absorbed. Thus, the intended purpose of repairing bone defects and reconstructing bone function^5^, ^6^ is achieved. Bone tissue engineering includes three basic elements: seed cells, scaffold materials and signalling factors.^7^, ^8^ The bone conductivity, biocompatibility, biodegradability, pore size, porosity and surface topography of the scaffolds have important effects on cell adhesion and growth and ultimately determine the effect on bone defect repair. Therefore, scaffolds play a vital role in bone tissue engineering.

There are two kinds of biomaterials used in tissue engineering scaffolds: natural materials and synthetic macromolecule materials. Natural materials mainly refer to natural materials or materials extracted from human or animal bodies. These materials include collagen, gelatin, cellulose, albumin, chitosan, alginate and so on. Synthetic macromolecule materials have good controllability and good processability, and their mechanical properties and degradation properties can be adjusted according to needs. Synthetic macromolecule materials can be processed into various required shapes. In recent years, many new materials have been used for tissue engineering, such as DNA nanostructures,^9^, ^10^ nucleic acids and analogs,^15^ nanosheets,^16^ carbon nanotubes^17^ and sodium montmorillonite film.^18^, ^19^ Therefore, synthetic macromolecule materials are also the most studied and widely used scaffolding materials in tissue engineering. Different electrospinning materials can achieve different electrospinning structures with different mechanical dexterity, biocompatibility and degradation rates_._ 21, 22 At the present, the scaffolds used in bone tissue engineering have some problems such as high cost, complex preparation and uncontrollable degradation; some scaffolds also cause inflammation; and foreign body reactions can even require surgical debridement and removal in some cases.^23^, ^24^ Poly(3‐caprolactone) nanocomposite scaffolds show great potential in tissue engineering.^25^ The natural amorphous copolymer poly(3‐hydroxybutyrate‐co‐4‐hydroxybutyrate) (P34HB), as a member of the bacterial biopolyesterpolyhydroxyalkanoate (PHA) family, is becoming a desirable candidate due to its remarkable mechanical properties, biocompatibility and bio‐degradability.^26^, ^27^ P34HB overcomes the issue of brittleness, which is associated with the narrow processing window of homopolymer poly‐3‐hydroxybutyrate (PHB),^29^ and shows an extension from 45% to 100% until the breaking point in accordance with 3‐8 mol% 4‐hydroxybutyrate. The favourable surface physicochemical properties, including hydrophilicity, surface free energy and polarity, that are determined by its high crystallization behaviour have attracted great interest for using P34HB as a carrier for the long‐term release of active biomolecules or as degradable implant materials. P34HB is the fourth generation member of the polyhydroxyalkanoate (PHA) family, and its performance is superior to that of the first three generations. It has been reported that the research on P34HB mainly focuses on the repair of cardiovascular tissue, cartilage and nerve tissue.^30^, ^31^ Our research group has performed many studies on P34HB in its early stage, and the results show that P34HB is a good scaffold material for tissue engineering.^34^, ^35^

Seed cells are required to come a wide range of sources, are easy to inoculate, demonstrate long‐term survive and have good stability. More importantly, the cells should be able to achieve rapid expansion in a specific environment and have strong osteogenic differentiation abilities.^36^, ^37^ Among the options, embryonic stem cells, bone marrow mesenchymal stem cells (BMSCs) and other stem cells are common seed cells. Stem cells have strong regenerative abilities and can be induced into cells with different functions under certain conditions. BMSCs are a kind of nonhaematopoietic stem cell that exists in bone marrow. BMSCs can support and regulate haematopoiesis in vivo and in vitro, and they can be distributed in many tissues and organs in vivo. BMSCs have multidirectional differentiation potential and can differentiate into osteoblasts, fibroblasts, reticular cells, adipocytes and endothelial cells, which makes them one of the most widely used seed cells.^38^, ^39^

The ideal scaffold materials in bone tissue engineering should have the following characteristics: three‐dimensional porous network that is conducive to cell growth; capability for nutrient transport and secreting metabolites; good biocompatibility and biodegradability, controllable degradation and absorption rates to adapt to the growth of cells or tissues in vivo and in vitro; chemical surface that is suitable for cell adhesion, proliferation and differentiation; and mechanical properties that match the requirements of the implanted tissues. Therefore, in this study, P34HB electrospun fibres were fabricated by electrospinning technology, and their properties were tested. The cytological behaviour of the scaffolds in vitro was examined. Next, we constructed an in vivo rat model of calvarial defects and implanted P34HB electrospun fibres by layer‐by‐layer paper‐stacking technology. Finally, the potential application of P34HB electrospun fibre scaffolds in bone tissue engineering was evaluated through experimental results.

## 2 MATERIALS AND METHODS

### 2.1 Fabrication of the P34HB electrospun fibres

First, 1 g macromolecule compound P34HB (kindly donated by Tsinghua University) was dissolved in a mixture of 20 ml chloroform and dimethylformamide (4:1, v/v; DMF; Sigma, St. Louis, MO, USA) and stirred for 12 hours at a speed of 60 r/min on a magnetic agitator. When the electric field force was greater than the surface tension of the liquid flow, the liquid flow was stretched into fibres and collected on aluminium foil. When the size and thickness of the P34HB electrospun fibres met our requirements, the electrospinning process was stopped and the P34HB electrospun fibres were removed from the aluminium foil to obtain the P34HB electrospun fibre scaffold.

### 2.2 Characterization of the P34HB electrospun fibre scaffold

The microstructure and porosity of the P34HB electrospun fibre scaffolds were observed by SEM (HITACHI S‐4800, Tokyo, Japan). Samples were fixed overnight with 5% glutaraldehyde, underwent a gradient dehydration with alcohol and lyophilized. Then, the samples were examined under an SEM. The hydrophilic angle of the scaffold was measured using a Cam 200 optical contact angle meter (KSV Instruments, Monroe, CT). The tensile properties of the scaffold were tested using a tabletop uniaxial testing instrument. The scaffolds were fixed on a universal capacity measuring instrument (Instron 5565, USA). The sample was stretched by a constant tensile force of 50 N until the sample was broken. All of the above tests were carried out six times.

### 2.3 Cytobehavioural analysis of the P34HB electrospun fibre scaffold

Bone marrow mesenchymal stem cells were extracted from 2‐week‐old transgenic mice enhanced with green fluorescent protein (GFP) using the whole bone marrow method. The mice were euthanized and disinfected, the femur and tibia were separated, and the metaphysis was dissected out. The bone marrow cavity was washed repeatedly with 10% FBS supplemented with low‐glucose DMEM (L‐DMEM). The obtained cells were cultured in an incubator at 37°C and 5% CO2. Half of the medium was exchanged at 48 h, and the entire medium volume was exchanged at 72 h. At 80 − 90% confluence, the cells were trypsinized and subcultured. After two subcultures, the purified BMSCs were obtained after the impurities and redundant tissues were removed.

After ultraviolet disinfection, the scaffolds were implanted into 6‐well plates and then inoculated with BMSCs at a density of 5‐10×10^4^ cells per mL. As a control, BMSCs were also inoculated into cultured plates without scaffold materials. The BMSCs of the two groups were observed with an Olympus IX 710 microscope (Olympus, Tokyo, Japan). For SEM, the scaffold was fixed with 3% glutaraldehyde for 1 hour, underwent a gradient dehydration with alcohol and lyophilized; the morphology of the BMSCs on the scaffold was then examined by SEM.

Biocompatibility and biosafety of the scaffold were detected by the cell counting assay kit‐8 (CCK‐8) (Dojindo, Japan). 10 mL of CCK‐8 solution was added to the both group, water‐soluble crystals can be formed after incubation in incubator at 37℃ for 1‐ 4 hours, and then, OD values at 450 nm can be determined by a microplate reader (VariOskan Flash 3001, Thermo, USA).

### 2.4 Bone defect formation and implantation in vivo

Thirty 12‐week‐old SD rats were used in this study, according to the method for establishing a model for critical‐sized calvarial defects.^40^ After anaesthetizing the rats with chloral hydrate through intraperitoneal injection at 0.3 − 0.35 mL/100 g, we made a 1.5 − 2 cm sagittal incision at the skull in the rats to separate the calvarium. Then, two full‐thickness defects of 5 mm were created symmetrically on both sides of the middle ridge using a trephine at 1500 rpm.

The experiment was divided into two time points: 4 weeks and 8 weeks. Each time point was divided into two groups: P34HB electrospun fibres group and blank control group, with 15 samples in each group. After the establishment of rat calvarial defects, we implanted the sterilized P34HB electrospun fibres layer by layer into the defect area until the defect area was filled. We call this specific method layer‐by‐layer paper‐stacking technology. After 4 and 8 weeks, that rats were executed and the skull specimens were obtained.

### 2.5 Radiological analysis

Acquired rat skull specimens were fixed in 4% paraformaldehyde for 2 days and were scanned by microcomputed tomography (*μ*CT scan, SCANCO Medical AG, Switzerland). VGStudio Max software was used to reconstruct the sample in three dimensions, and views in all levels were reconstructed to observe the regenerated calvarium bone in detail. The red‐green colour pattern is used to quantitate the three‐dimensional images, and the red‐green pattern represents the density (Hounsfield unit, Hu) of the tissue from high to low.

### 2.6 Histological evaluation

The samples were histologically assessed after the micro‐CT examination. Samples were immersed in 15% EDTA‐buffered saline solution for 15 days in order to be decalcified, and then, the samples were treated with a series of graded alcohol baths for dehydration, treated with xylene, embedded in paraffin and cut into 5 μm thick slices. The slices were dewaxed in xylene I and II for 5 minutes, then hydrated in an alcohol gradient and incubated in deionized water for 5 minutes; the nuclei were stained with haematoxylin for 2 minutes, and the slices were rinsed with tap water for 15 minutes and then differentiated with 75% ethanol hydrochloride for 2 seconds. Half of the slices were dyed with acidified with eosin ethanol (HE) for 30 seconds, and half of the slices were stained with Masson's trichome stain for several seconds; all of the slices were dehydrated in an alcohol gradient followed by xylene III and IV for 5 minutes. Finally, the stained slices were sealed with neutral gum. The specimens were scanned with a tissue scanner (Aperio, ScanScope XT).

### 2.7 Statistical analysis

Three or more independent experiments were performed, and each experiment was run at least three times. Statistical analysis was performed with SPSS 19.0 (SPSS, Chicago, IL, USA). All data were expressed as the mean±standard deviation. Data were analysed by one‐way analysis of variance (ANOVA), followed by the Student‐Newman‐Keuls test. *P* < 0.05 was considered to be statistically significant.

## 3 RESULTS

### 3.1 Characteristics of the P34HB electrospun fibre scaffold

**Figure 1:**
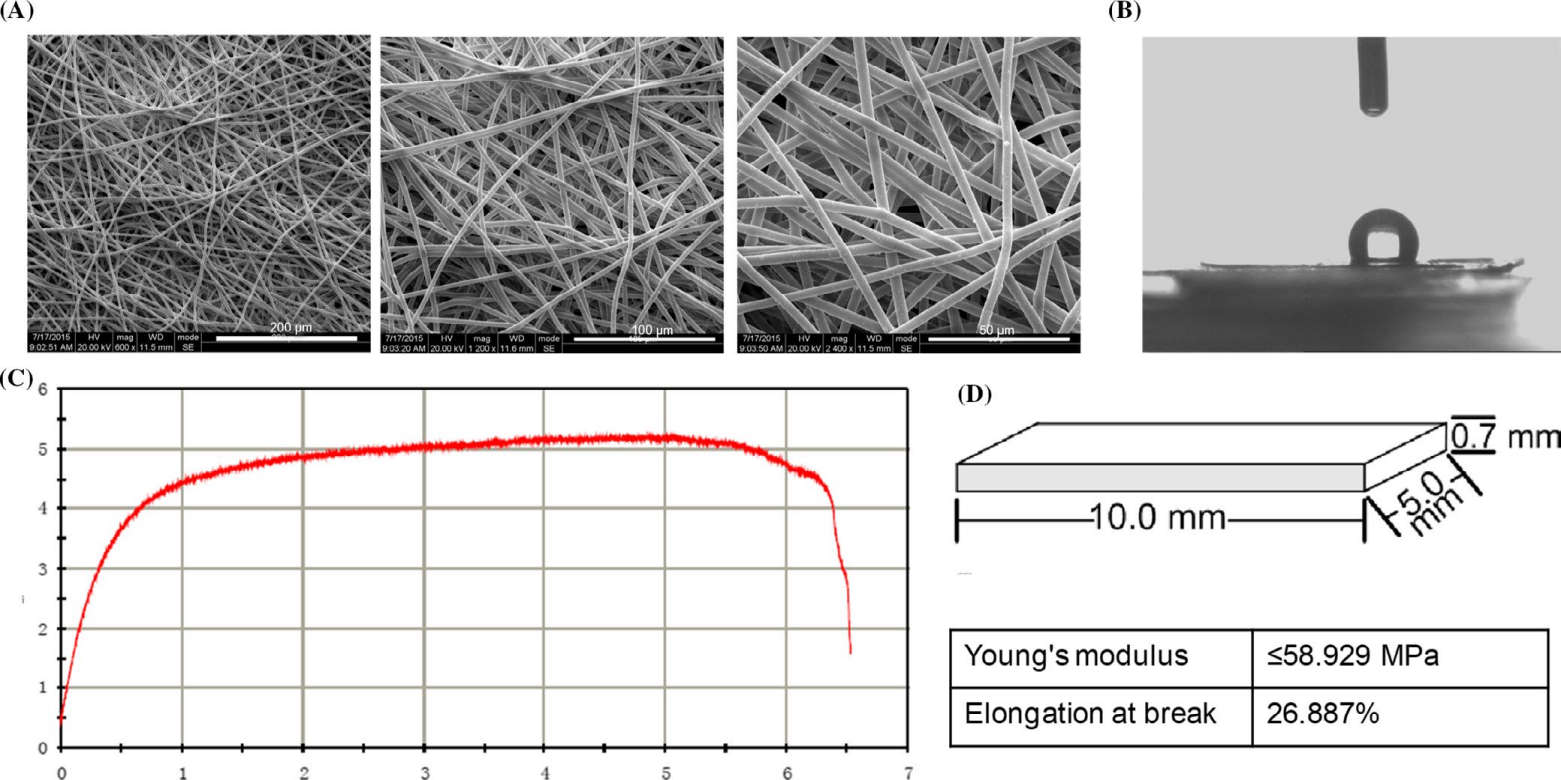
Characterization of P34HB electrospun fibres scaffold. A, Morphology of the scaffold evaluated by SEM (n = 3). The structure of the scaffold is interwoven by a number of fibres, which are arranged randomly and have many holes. B, Measurement of the water contact angle wetting behaviour of a water droplet on the scaffold (n = 3), the contact angle was determined at 116.5 ± 2.3°. C, Mechanical parameters determined from slices of the scaffold (10.0 × 5.0 × 0.7 m^3^) (n = 3). The maximum strength the scaffold could bear was 5 N, and the scaffold can be stretched to ~6 mm before breaking. D, The mechanical parameters of fibres were measured. The Young's modulus of randomly selected fibres was approximately 58.929 MPa, and the elongation at the breaking point was 26.887%

The morphologies of the scaffolds were characterized by SEM (Figure 1A). The SEM results show that the structure of the scaffold is interwoven by a number of fibres, which are arranged randomly and have many holes. The contact angle of the scaffold (Figure 1B) was determined to be 116.5 ± 2.3°. In the tension test, the maximum strength the scaffold could bear was 5 N, and the scaffold can be stretched to ~6 mm before breaking (Figure 1C). The mechanical parameters of the fibres were measured. The Young's modulus of randomly selected fibres was approximately 58.929 MPa, and the elongation at the breaking point was 26.887%.

### 3.2 In vitro cellular assays

**Figure 2:**
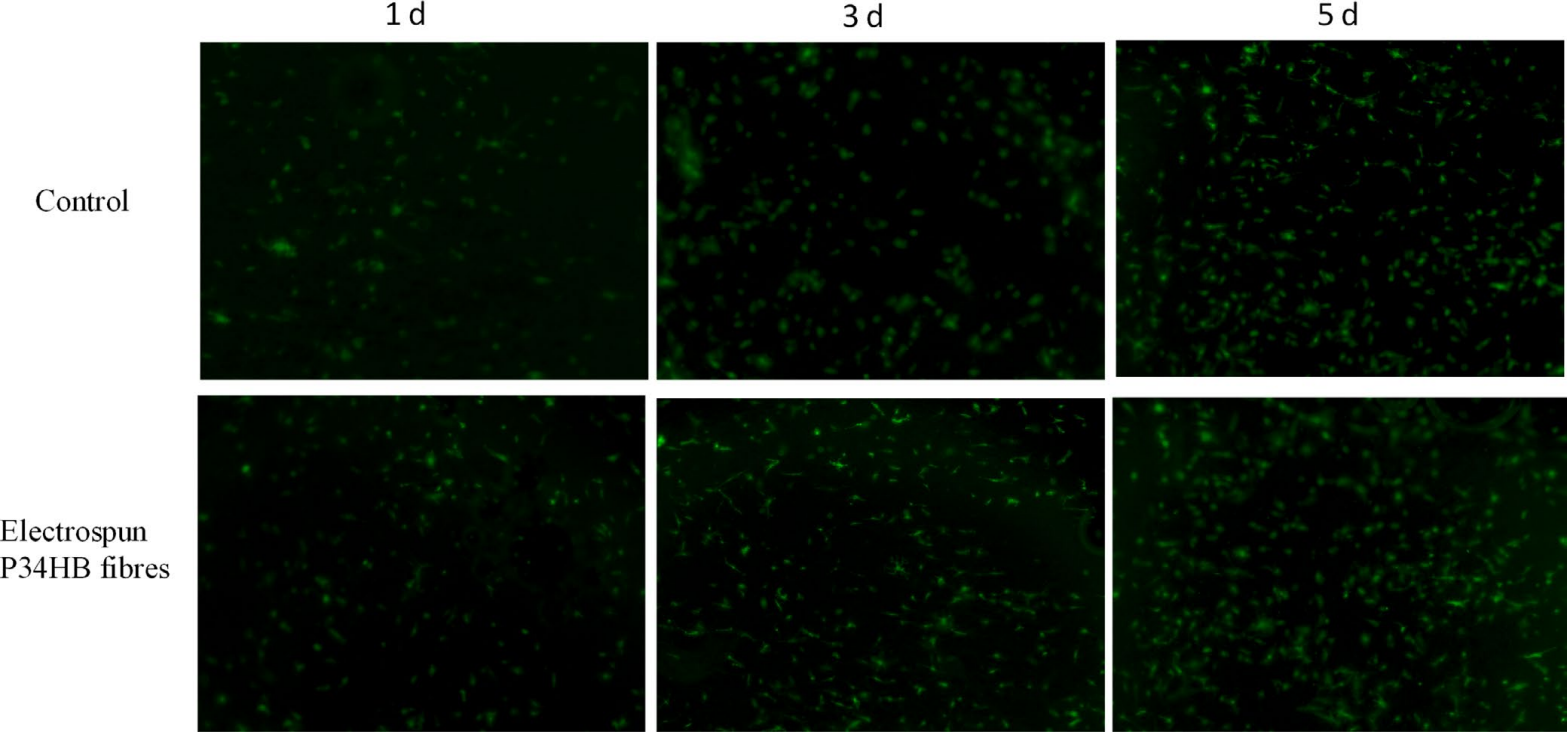
Compared with the control group, P34HB electrospun fibres scaffold can promote cell adhesion and proliferation. As shown by microscope observation, bone marrow mesenchymal stem cells (BMSCs) attached to the scaffold after seeding. As time goes on, the cell spreading area was the broader and the number of BMSCs was significantly increased in comparison to the control group

**Figure 3:**
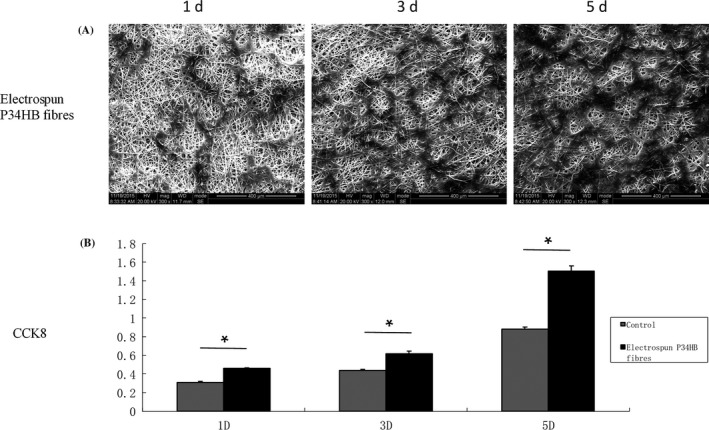
In vitro cell behaviour of the P34HB electrospun fibres scaffold. A, Cell morphologies of bone marrow mesenchymal stem cells (BMSCs) on the fibres, as shown by SEM (n = 3). BMSCs were seeded, attached, spread and proliferated within 5 days. B, Cell proliferation of BMSCs on the fibres and Petri dishes (n = 3). The results show that the proliferation rates within 5 days were significantly higher on the fibres than that found on the Petri dishes

The morphology of the BMSCs on the P34HB electrospun fibres was detected by fluorescence microscopy (Figure 2). The BMSCs successfully attached to the fibres after seeding, and compared with the control group, the number of cells in the scaffold group was higher. The BMSCs seeded 1, 3 and 5 days on the P34HB electrospun fibres showed changes in cell morphology and numbers by SEM (Figure 3A). Over time, the BMSCs gradually covered the P34HB electrospun fibres and penetrated into the pores. The CCK‐8 assay results (Figure 3B) showed that after days 1, 3 and 5, the number of BMSCs on the scaffold group was significantly higher than that of the control group, and the cell proliferation performance was stronger than that of the control group.

### 3.3 In vivo bone repair of calvarial defects

**Figure 4:**
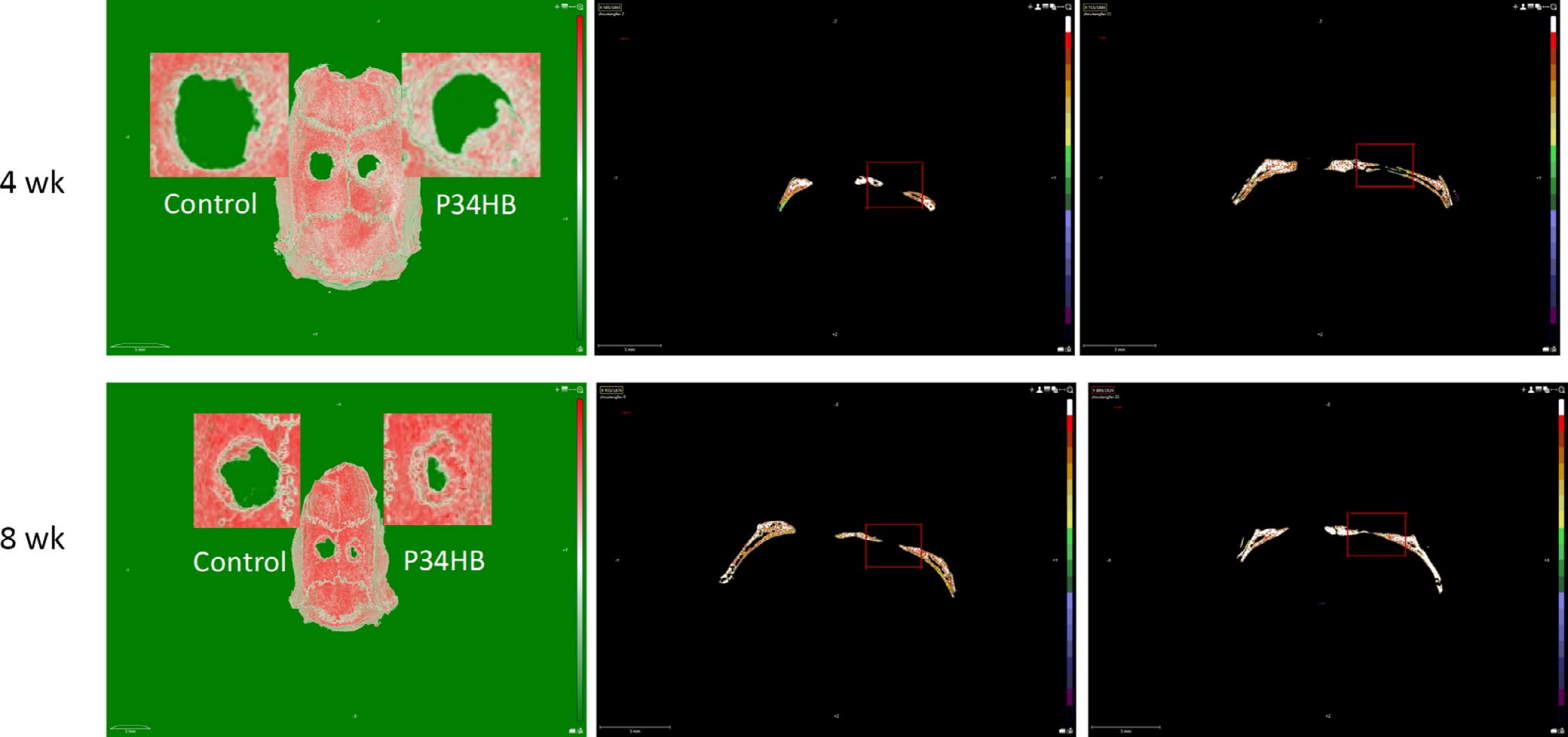
Radiographical analysis of bone formation. Compared with the control group, the effect of the scaffold group on defect repair was better at both time points. There were more green areas in the control group and more red areas in the scaffold group, where green represents low mineralized fibrous tissue and red represents mineralized bone. At 8 weeks, the best results were achieved in the scaffold group, and the defect area was basically covered by red. Moreover, new bone‐like tissue grew from the periphery of the defect area into the centre

Three‐dimensional reconstructions of the calvarial defect area were performed by micro‐CT (Figure 4A). Compared with the control group, the effect of the scaffold group on defect repair was better at both time points. There were more green areas in the control group and more red areas in the scaffold group, where green represents low mineralized fibrous tissue and red represents mineralized bone. At 8 weeks, the best results were achieved in the scaffold group, and the defect area was basically covered by red. Moreover, new bone‐like tissue grew from the periphery of the defect area into the centre.

**Figure 5:**
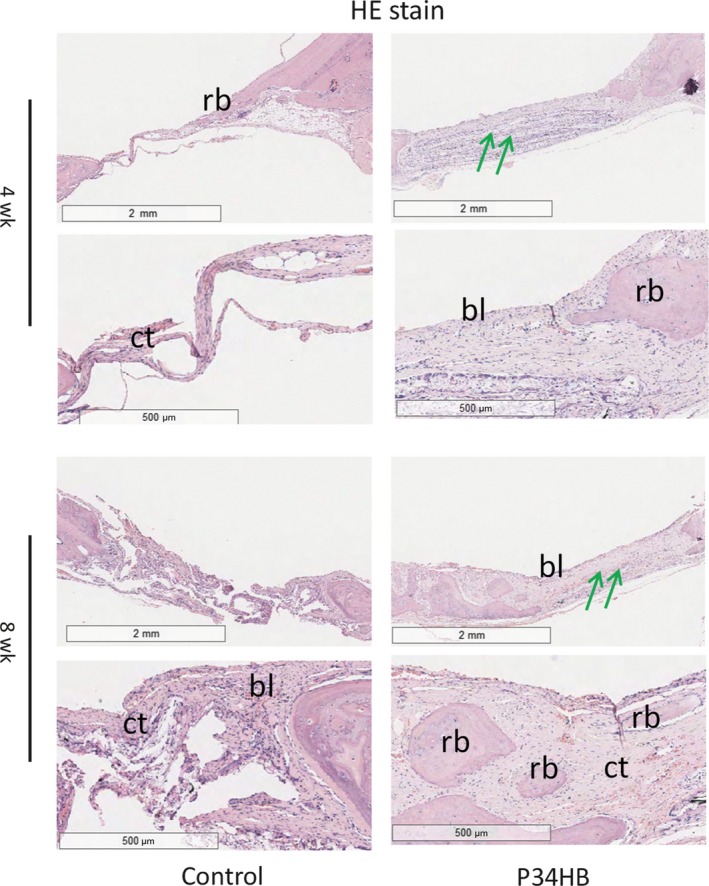
HE staining of cross‐sections of repaired calvarial defects at 4 and 8 weeks. The results of each group at 8 weeks were better than those at 4 weeks, and the P34HB group results were better than the results of the control group. In the control group, we observed only a large amount of connective tissue and a small amount of bone‐like tissue, 8 weeks after implantation, the P34HB scaffolds had a large number of new bone islands, some regenerated bone and bone‐like tissue had replaced the gradually degraded scaffold material, and the bony bridge was almost fully linked with clear, mature bone structures (bl, bone‐like tissue; ct, connective tissue; rb, regenerated bone; green arrows, layered scaffold structures)

**Figure 6:**
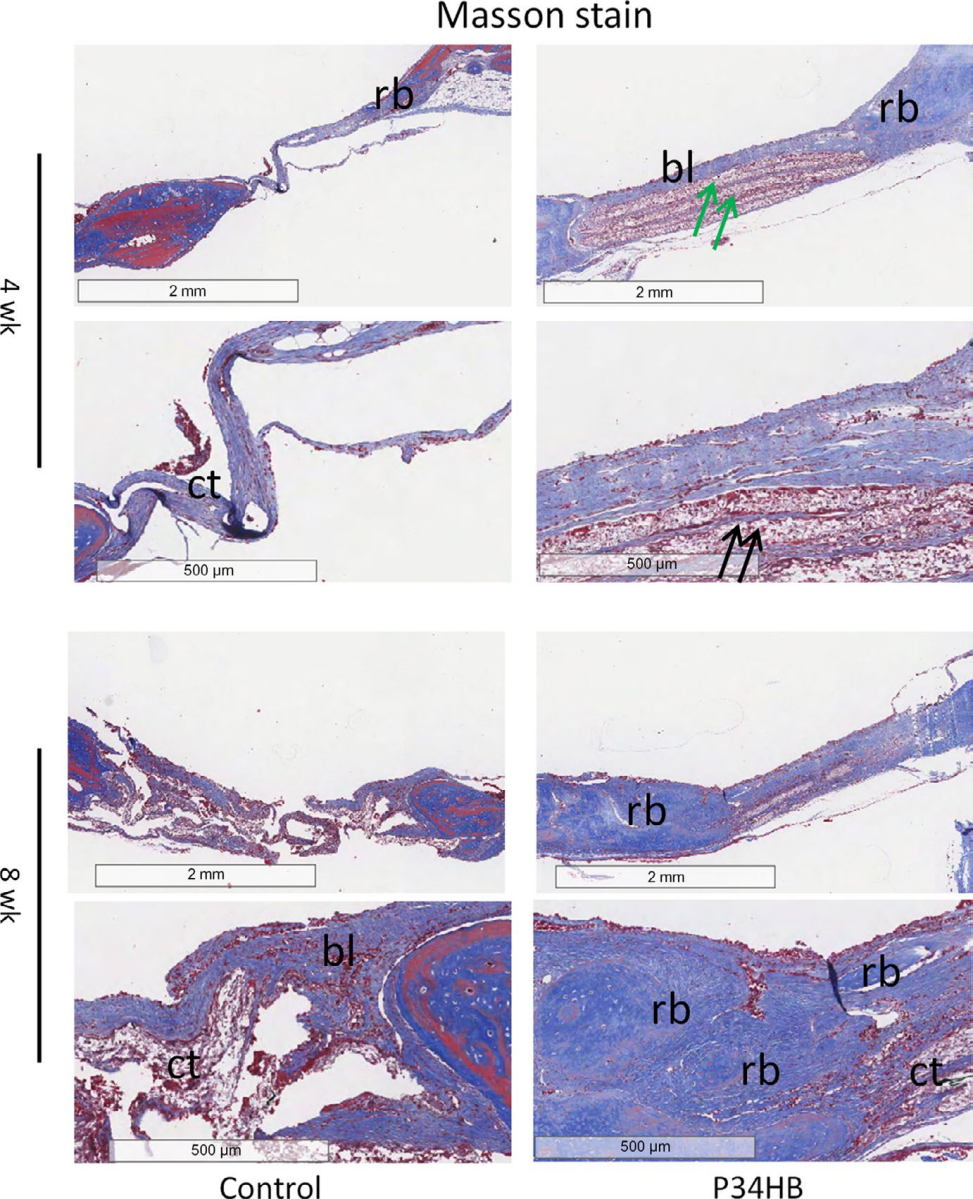
The results of Masson's trichome staining were consistent with those of HE staining. The P34HB group results were better than the results of the control group at the two time points. In the control group, we observed only a large amount of connective tissue and a small amount of bone‐like tissue. The best results were obtained after 8 weeks of implantation when a large number of new bone islands could be seen, some regenerated bone and bone‐like tissue had replaced the gradually degraded scaffold material, and the bony bridge was almost fully linked with clear, mature bone structures (bl, bone‐like tissue; ct, connective tissue; rb, regenerated bone; green arrows: layered scaffold structures; black arrow, blood vessels)

Eosin ethanol staining (Figure 5) showed the boundary between the neoplastic tissue and the surrounding original tissue in the defect area. The results of each group at 8 weeks were better than those at 4 weeks, and the P34HB group results were better than the results of the control group. In the control group, we observed only a large amount of connective tissue and a small amount of bone‐like tissue, 8 weeks after implantation, the P34HB scaffolds had a large number of new bone islands, some regenerated bone and bone‐like tissue had replaced the gradually degraded scaffold material, and the bony bridge was almost fully linked with clear, mature bone structures. The results of Masson's trichome staining were consistent with those of HE staining (Figure 6). The P34HB group results were better than the results of the control group at the two time points. In the control group, we observed only a large amount of connective tissue and a small amount of bone‐like tissue. The best results were obtained after 8 weeks of implantation when a large number of new bone islands could be seen, some regenerated bone and bone‐like tissue had replaced the gradually degraded scaffold material, and the bony bridge was almost fully linked with clear, mature bone structures.

## 4 DISCUSSION

Bone defects, resulting from trauma, abnormalities, osteomyelitis, necrosis and tumours, have posed tremendous challenges for clinical management. Traditional clinical methods for repair involve allo‐/autografts, artificial materials and distraction osteogenesis, which may have provided positive results, but still have obvious limitations, such as donor morbidity, inferior healing capability, immune complications and cosmetic concerns. In 1995, Grane^41^ systematically put forward the concept, research methods, research status and development prospects of bone tissue engineering, which attracted the attention of scholars. The scientific field of bone tissue engineering uses the principles and methods of tissue engineering to develop bone substitutes with the ability to repair bone defects. The basic principle is to construct a scaffold material‐seed cell‐growth factor complex in vitro, then transplant the complex to the bone defect, for material absorption and tissue growth, slowly replace the tissue‐engineered bone with self‐bone tissue, and ultimately form the shape of bone and achieve functional repair. Although intensive research has focused on the developmental biology and regeneration of bone tissue, a diverse plethora of biomaterials has been developed for this purpose. Bone regeneration is still suboptimal with disadvantages such as lack of a layered structure, mechanical mismatch with native bone and inadequate integration between the native tissue and the implanted scaffold. Therefore, the key step of bone tissue engineering research is to develop scaffolds for cell transplantation that will guide new bone growth while substituting for the extracellular matrix.

Electrospinning technology has been rapidly developing in recent years. Electrospinning can produce nanofibres by simple and effective methods. This technology may play a vital role in the fields of medicine, chemistry, optoelectronics, energy and food engineering.^42^, ^43^ The applications of electrospinning in medicine are mainly for constructing various types of scaffolds for tissue engineering that can be constructed with fibres that have diameters ranging from tens of nanometres to micrometres, which is a result that other technologies cannot achieve.^45^ The construction of three‐dimensional scaffolds for tissue engineering provides a new idea and method for bone defect.^46^, ^47^

The scaffold material is the main component of bone tissue engineering and performs the function of extracellular matrix, including providing a suitable microenvironment for the repair of tissue cells, resisting the pressure from surrounding tissues, and temporarily performing some load‐bearing functions. P34HB has been shown to have a wide range of physical properties ranging from high crystallinity to elastic behaviour depending on the mol% of the 4HB monomer. Crystallization plays an important role in changing the hydrophilicity, surface free energy and polarity and therefore affects cell attachment and proliferation. BMSCs are considered to be ideal seed cells because of their multidirectional differentiation, strong proliferation and low immunogenicity. In our study, the performance and behaviour of BMSCs on P34HB electrospun fibres were tested. The scaffold had a three‐dimensional porous network, and the cell adhesion and proliferation of the scaffold group were high relative to that of the Petri dish control group due to the good physical properties of the P34HB electrospun fibres. The in vitro cell experiments provided direct support for the next implant study.

A successful implant biomaterial should meet important requirements, including the controllable degradation of the implant and the integration between the native tissues and the P34HB electrospun fibre scaffold. The degradation products of P34HB based on PHAs were oligo‐HAs including oligo(3‐hydroxybutyrate) (OHB) and oligo(3‐hydroxybutyrate‐co‐3‐hydroxyhexanoate) (OHBHHx). Previous studies on murine beta cells have directly confirmed that these degradation products have positive effects on cell growth. However, the diffusion of oxygen and nutrients in the 3D scaffolds loaded with seed cells is often insufficient, and it is difficult to reach the centre of the 3D scaffolds. It is difficult to excrete the cell secretions from the centre of the scaffold. The signal transduction between the cells and the scaffold or between cells and other cells is not smooth enough. The spatial chemical gradient cannot be established, which means that the microenvironment of the cell tissue cannot be established. This gap will directly affect the application and development of tissue engineering.^48^, ^49^ To solve these problems, a team led by Professor Whitesides^50^ of Harvard University proposed a framework structure for membrane layer‐by‐layer assembly and demonstrated that if the scaffold material was thin enough and permeable enough, oxygen and nutrients can smoothly diffuse to the centre of scaffold and maintain a certain spatial and temporal chemical gradient, that is, the cell microenvironment in tissue. In our study, critical‐sized calvarial defects were created, and the scaffold was implanted by layer‐by‐layer paper‐stacking technology. The results showed that the P34HB electrospun fibres could promote bone healing.

There are still some limitations in the current study. First, the investigation of whether the newly repaired tissue is fibrous osteoid tissue requires long‐term follow‐up, although the micro‐CT and histological staining have confirmed the markers of bone in the newly formed tissues. Second, the time during which the P34HB electrospun fibres could entirely degrade and the new bone could be completely formed should be confirmed. Third, whether the mechanical properties of the newly formed tissues are close to those of native bone also needs to be further investigated.

## 5 CONCLUSIONS

In this study, a P34HB electrospun fibre scaffold was fabricated, and its mechanical properties, cell behaviour and ability to repair calvarial defects were determined in vitro and in vivo. The results showed that the scaffold had a three‐dimensional pore structure, which was conducive to cell entry and material transport; had mechanical properties to meet physiological needs; had good plasticity, which could be tuned according to the defect situation; had good biocompatibility, which did not cause immune rejection, toxicity effects, teratogenicity or carcinogenesis; had surface microstructures that were conducive to cell adhesion, proliferation and growth; and was degradable and eventually replaced by autologous regenerated tissue. Our findings demonstrate that the P34HB electrospun fibre scaffolds satisfy the characteristics of ideal scaffolds and can be an ideal scaffold for bone tissue engineering with great application potential.
